# A Rare Case of Isolated Diverticular Disease of the Appendix Combined With Uncomplicated Appendicitis

**DOI:** 10.7759/cureus.75575

**Published:** 2024-12-12

**Authors:** Youssef Assaad Ahmed, Rawan Manna, Priya Thomas, Ali Yasen Mohamedahmed, James Eccersley

**Affiliations:** 1 General Surgery, Queen’s Hospital Burton, University Hospitals of Derby and Burton NHS Trust, Burton on Trent, GBR; 2 Histopathology, University Hospitals of Derby and Burton NHS Trust, Burton on Trent, GBR

**Keywords:** appendictis, diverticular disease of the appendix, diverticulitis, laparoscopic treatment, right iliac fossa

## Abstract

The differential diagnoses for patients presenting with right iliac fossa pain are broad, with appendicitis almost always on the top of the list. Although rare, diverticulosis of the appendix, complicated by inflammation, should be considered in these patients. We report a case of a middle-aged female with right iliac fossa pain with a high inflammatory marker. CT of the abdomen and pelvis showed uncomplicated appendicitis. The patient underwent a diagnostic laparoscopy, which confirmed an inflamed appendix and three small nodules in the body of the appendix. An appendectomy was performed, and histopathology showed diverticulosis of the appendix with appendicitis. She recovered uneventfully, and the follow-up colonoscopy was normal. This report illustrates the complexities associated with diverticular disease of the appendix and its potential to mimic appendicitis and highlights the importance of histopathology examination in these cases.

## Introduction

Diverticular disease of the appendix, although rare, presents a unique clinical challenge, particularly when associated with appendicitis. Diverticular disease in the appendix was first described by Kelynack in 1893 [1]; the incidence of diverticula of the appendix found in appendectomy specimens ranges from 0.004% to 2.1% [2]. Appendiceal diverticulitis may present similarly to acute appendicitis; however, it is more prevalent in males in their fifth decade of life and has a slightly higher morbidity rate compared to appendicitis alone. Moreover, it is more common in patients with Hirschsprung’s disease and cystic fibrosis [3]. We discuss a case of a middle-aged female with diverticular disease of the appendix with features of acute appendicitis.

## Case presentation

The patient was a middle-aged female who was referred to the surgical department with a two-week history of gradually worsening central abdominal pain that had shifted to her right iliac fossa three days before her emergency department visit. She reported no associated changes in bowel habits, temperature spikes, nausea, or vomiting. She also denied any significant past medical conditions or recent travel history. Initial abdominal examination revealed rebound tenderness in the right iliac fossa. Blood investigations showed a white cell count (WCC) of 13.3 x10^9^/L (normal range: 4.5-11.0 ×10^9^/L) and a C-reactive protein (CRP) of 38 mg/L (normal range: ≤1 mg/L), with the remaining tests within normal limits.

**Figure 1:**
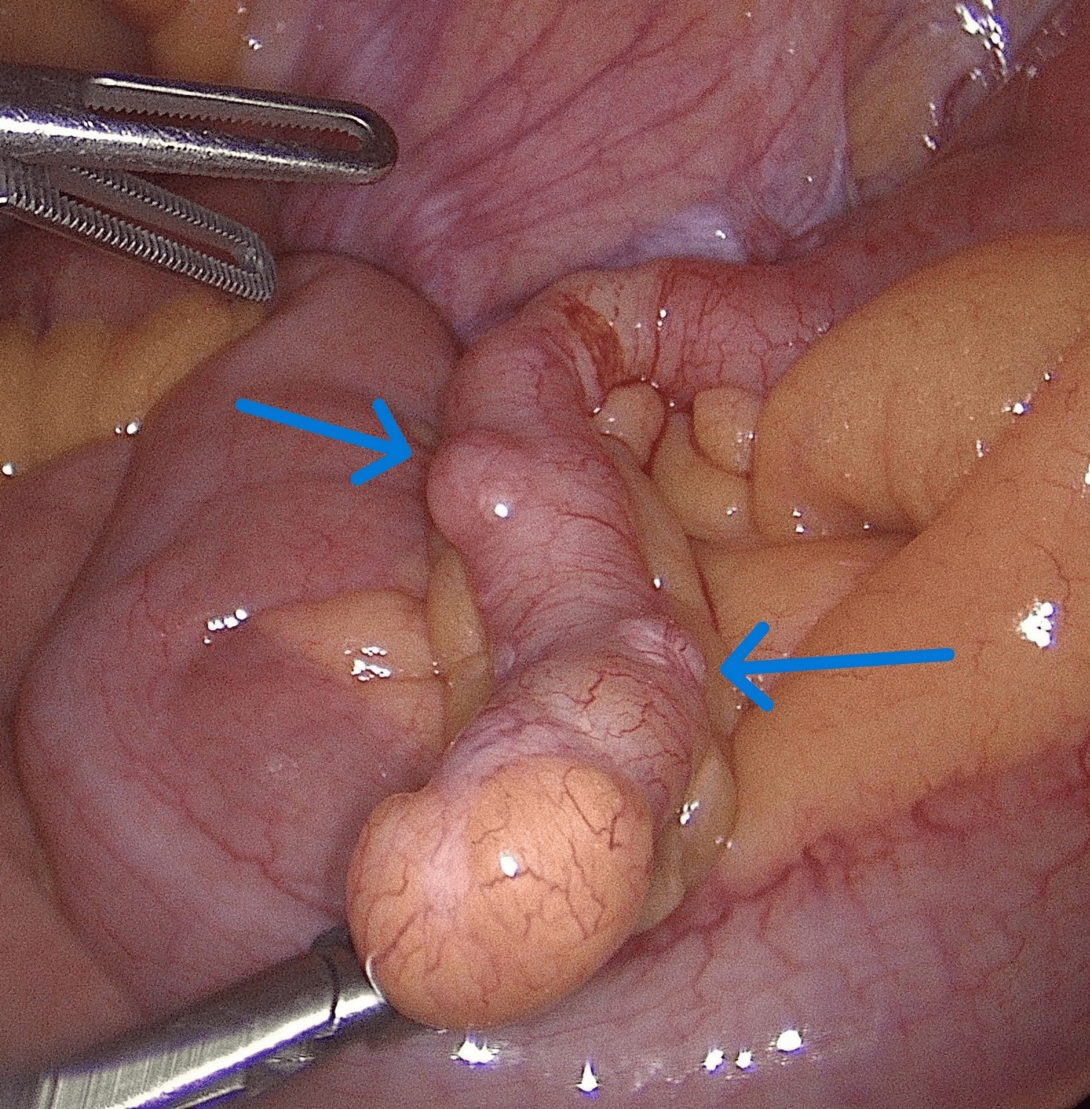
Intraoperative findings during diagnostic laparoscopy Blue arrows point to two small nodules in the body of the inflamed appendix

Additionally, a CT of the abdomen and pelvis (CTAP) suggested early and uncomplicated acute appendicitis. Following initial treatment with intravenous fluids and antibiotics, she underwent a diagnostic laparoscopy, which showed an uncomplicated inflamed appendix with three nodules in the body of the appendix, each less than 8 mm in size (Figure 1).

**Figure 2:**
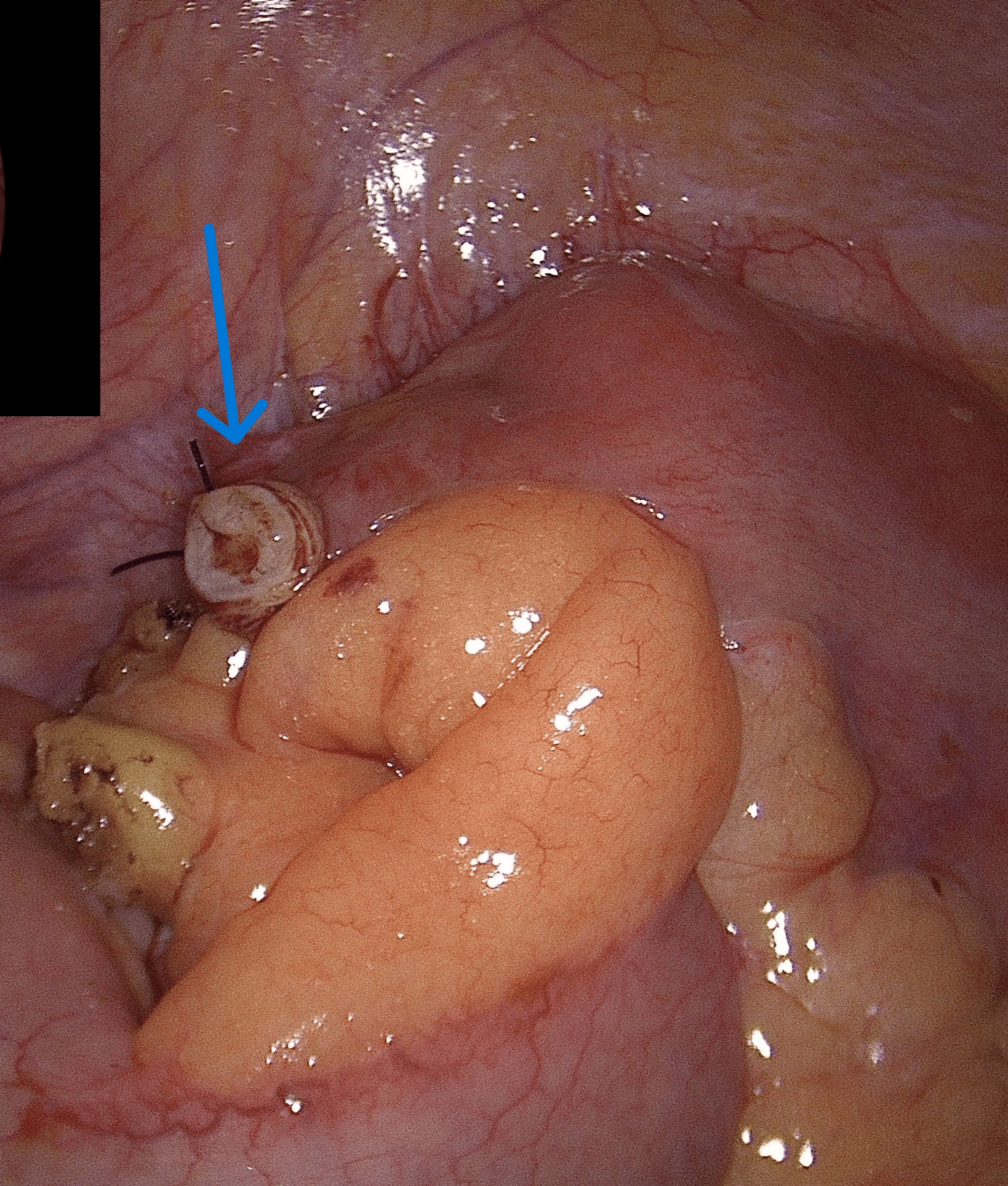
Appendicectomy procedure (labelled with blue arrow)

The rest of the abdominal organs, including the liver, were normal. Subsequently, the appendix was removed using three endoloops with the entire mesoappendix as the nature of the nodules was unclear (Figure 2).

**Figure 3:**
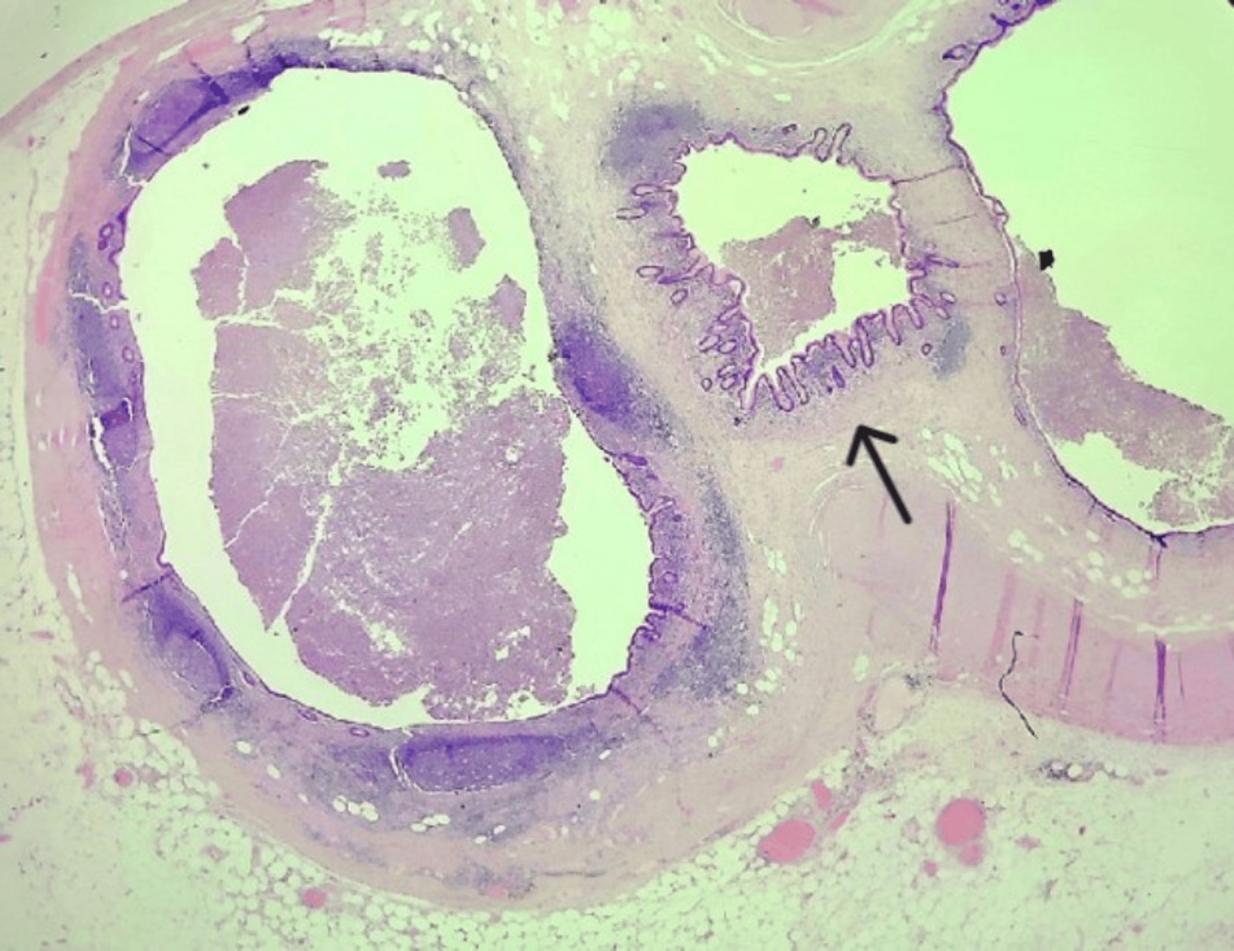
Longitudinal section of the appendix tip showing acquired appendiceal [pseudo]diverticulosis Haematoxylin and eosin staining, 20x magnification. The mucosa and submucosa are herniating through the weakened muscularis propria (black arrow)

The patient was discharged home on postoperative day one, following an uneventful recovery. Histopathological sections from the appendix specimen demonstrated acute appendicitis with transmural acute inflammation and mucosal ulceration. In addition, diverticular disease was observed in the body of the appendix, and no neoplasia or infectious organisms were identified (Figure 3).

The patient underwent a follow-up colonoscopy, which showed a normal colon without any features of diverticular disease.

## Discussion

Appendiceal diverticula are outpouchings of the mucosal layer through the muscular wall of the appendix. The two main subtypes of appendicular diverticulosis are congenital and acquired [4]. The congenital subtype, a true diverticulum, involves all layers of the appendicular wall. Moreover, congenital appendicular diverticulosis has been linked to conditions such as cystic fibrosis, Patau syndrome (trisomy 13) and trisomy 15 [5]. On the other hand, acquired appendicular diverticula, which are more commonly observed, are false diverticula and involve only the mucosa and submucosa. Additionally, acquired appendicular diverticulosis can occur secondary to increased pressure within the least resistant points in the lumen of the appendix, leading to an outpouching of the mucosal layers through to the muscularis propria layer along the antimesenteric borders [3-5].

It can be challenging to distinguish the symptoms of appendicular diverticulitis from those of acute appendicitis, and, usually, the diagnosis is only established by the histology study of the specimen [6]. However, certain distinguishing features of the condition include longer duration of symptoms and intermittent pain episodes that may precede the acute episode [4]. It is worth mentioning that the duration of abdominal pain in the presented case was almost two weeks, which is not a typical presentation in uncomplicated appendicitis.

Phillips et al. have classified appendicular diverticulosis into four types according to the pathological changes of the diverticulum and the appendix [7]. The histological findings of the reported case - simple inflammation of the appendicular tissues and non-inflamed diverticula - align with type four. Types one and two are primary acute diverticulitis and acute diverticulitis secondary to acute appendicitis, respectively. The third type is simple diverticula without inflammation; a fifth type has also been identified: chronic peri-diverticulitis with acute appendicitis. Appendicular diverticular disease may increase the risk of appendicular neoplasia [8]; however, the histopathology examination of this case revealed no neoplastic cells. The morphological appearance of the diverticula in the appendix during laparoscopy raises suspicion for appendicular tumours, and the differential diagnosis includes carcinoid and non-carcinoid tumours, pseudomyxoma peritonei (PMP) and adenocarcinoid tumours.

CTAP has low sensitivity for detecting appendicular diverticulosis, as less than 7% of diverticulitis of the appendix is diagnosed on CTAP alone [9]. Interestingly, Kubota et al. have reported a case of appendicular diverticulitis diagnosed on an ultrasound scan [10]. The reported features included thickened and echogenic appendicular wall layers due to air in the diverticula. A case series of 24 cases concluded that the radiological features of appendicular diverticulitis include peri-appendiceal extra-luminal loculated fluid, peri-appendiceal fat stranding, and a larger appendix diameter [11]. These radiological features often resemble and are misinterpreted as acute appendicitis.

Postoperative recovery in our patient was uneventful, and she was discharged with no complications. The follow-up colonoscopy, which revealed no colonic diverticulosis, is particularly noteworthy. It suggests that the diverticular disease was localised to the appendix, which is a rare occurrence as most diverticular disease is associated with the colon [4-8]. This case emphasises the importance of follow-up investigations in patients with diverticular disease of the appendix, to rule out the presence of diverticulosis elsewhere in the gastrointestinal tract. Prophylactic appendicectomy has been recommended when appendicular diverticulosis is discovered incidentally during abdominal scans and diagnostic procedures [4], as these patients are at higher risk of developing appendicitis (60%). Moreover, patients with acute appendicular diverticulitis are four times more likely to suffer from a perforation compared to those with acute appendicitis, leading to a 30-fold increase in mortality [3,6].

## Conclusions

This case report illustrates the complexities associated with diverticular disease of the appendix and its potential to mimic appendicitis. CTAP scans cannot be solely relied on to diagnose diverticular disease in the appendix, and usually, the diagnosis is reached after the histopathological examination, ruling out coexistent neoplasia. Clinicians, including surgeons, pathologists, and radiologists, should include appendiceal diverticular disease in the differential diagnosis of appendicitis.
